# ACR TI-RADS and ATA US scores are helpful for the management of thyroid nodules with indeterminate cytology

**DOI:** 10.1186/s12902-019-0429-5

**Published:** 2019-10-29

**Authors:** Thayse Lozovoy Madsen Barbosa, Cleo Otaviano Mesa Junior, Hans Graf, Teresa Cavalvanti, Marcus Adriano Trippia, Ricardo Torres da Silveira Ugino, Gabriel Lucca de Oliveira, Victor Hugo Granella, Gisah Amaral de Carvalho

**Affiliations:** 1 Department of Endocrinology and Metabology, Federal University of Paraná– Brazil, Av. Agostinho Leão Júnior, 285, Alto da Glória, Curitiba, PR 80.030-110 Brazil; 2 Department of Endocrinology, Clinical Hospital of the Federal University, Av. Agostinho Leão Júnior, 285, Alto da Glória, Curitiba, PR 80.030-110 Brazil; 3 Department of Pathology, Clinical Hospital of the Federal University of Paraná, Rua General Carneiro, 181, Alto da Glória, Curitiba, PR 80.060-900 Brazil; 4 Radiology, Rua Marechal Deodoro, 503, Curitiba, PR Brazil; 5 Medical School, Federal University of Paraná, Curitiba, Brazil

**Keywords:** Thyroid nodules, Indeterminate, ACR TI-RADS, American Thyroid Association (ATA) guidelines, Ultrasonography

## Abstract

**Background:**

Cytologically indeterminate thyroid nodules currently present a challenge for clinical decision-making. The main aim of our study was to determine whether the classifications, American College of Radiology (ACR) TI-RADS and 2015 American Thyroid Association (ATA) guidelines, in association with The Bethesda System for Reporting Thyroid Cytopathology (TBSRTC), could be used to stratify the malignancy risk of indeterminate thyroid nodules and guide their clinical management.

**Methods:**

The institutional review board approved this retrospective study of a cohort of 140 thyroid nodules in 139 patients who were referred to ultrasound-guided fine-needle aspiration cytology (FNAC) from January 2012 to June 2016 with indeterminate cytological results (44 Bethesda III, 52 Bethesda IV and 44 Bethesda V) and in whom pre-FNAC thyroid US images and histological results after surgery were available. Each included nodule was classified by one radiologist blinded to the cytological and histological diagnoses according to the ACR TIRADS scores and the US patterns as recommended in the 2015 ATA guidelines. The risk of malignancy was estimated for Bethesda, TI-RADS scores, ATA US patterns and their combination.

**Results:**

Of the 140 indeterminate thyroid nodules examined, 74 (52.9%) were histologically benign. A different rate of malignancy (*p* < 0.001) among Bethesda III, IV and V was observed. The rate of malignancy increased according to the US suspicion categories (p < 0.001) in both US classifications (TI-RADS and ATA). Thyroid nodules classified as Bethesda III and the lowest risk US categories (very low, low and intermediate suspicion by ATA and 2, 3 and 4a by TI-RADS) displayed a sensitivity of 95.3% for both classifications and a negative predictive value of 94.3 and 94.1%, respectively. The highest risk US categories (high suspicion by ATA and 4b,4c and 5 by TI-RADS) were significantly associated with cancer (odds ratios [ORs] 14.7 and 9.8, respectively).

**Conclusions:**

Ultrasound classifications, ACR TI-RADS and ATA guidelines, may help guide the management of indeterminate thyroid nodules, suggesting a conservative approach to nodules with low-risk US suspicion and Bethesda III, while molecular testing and surgery should be considered for nodules with high-risk US suspicion and Bethesda IV or V.

## Background

Thyroid nodules (TNs) are very common in clinical practice, with a prevalence of up to 68% by US in the general population [1]. The challenge of the clinician is to exclude thyroid cancer, which occurs in a small subgroup of nodules (~ 10%) [2]. Establishing a differential diagnosis is essential to avoid unnecessary surgeries in asymptomatic benign nodules and delayed diagnosis and treatment for malignant lesions. Fine-needle aspiration cytology (FNAC) is an accurate and cost-effective tool in which benign and malignant diagnoses carry malignancy risks of approximately < 5 and > 96%, respectively [3]. Unfortunately, approximately 25% of all biopsies have indeterminate cytology [4]. The Bethesda System for Reporting Thyroid Cytopathology (TBSRTC) classifies indeterminate cytological results into 3 of 6 categories: Bethesda III - Atypia of Undetermined Significance/Follicular Lesion of Undetermined Significance (AUS/FLUS), Bethesda IV - Follicular Neoplasm/Suspicious for a Follicular Neoplasm (FN/SFN) and Bethesda V - Suspicious for Malignancy (SM) [5]. For indeterminate TNs, molecular tests are proposed in an attempt to refine the preoperative diagnosis to reduce the rate of diagnostic surgeries [6, 7]. However, the significant cost of the molecular markers and their unavailability in all health-care centers make their use in clinical practice unfeasible.

Ultrasound (US) has a central role in the evaluation of TNs that are eligible for FNAC [8, 9]. Two of the most widely known thyroid nodule US classifications are the Thyroid Imaging Reporting and Data System (TI-RADS) and the 2015 American Thyroid Association guideline (2015 ATA) [2]. Initially, TI-RADS was reported by Horvath et al. [10], as well as a subsequent proposal by Park et al. [11] and Kwak et al. [12]. In 2017, the American College of Radiology (ACR) recommended a point system for the assessment of imaging TNs. Points are assigned based on 5 ultrasound features and the sum determines the ACR TI-RADS classification of the nodule [13]. The 2015 ATA guidelines classified the TNs into five patterns according to the combination of US features and each pattern had an estimated risk of malignancy (Table 1). According to the size and TI-RADS score or ATA pattern, the nodule was referred for FNAC or follow-up. Features that raise the suspicion for thyroid cancer are marked hypoechogenicity, microcalcifications, irregular margins, and taller-than-wide shape [12, 14–17]. The performance of 2017 ACR TI-RADS was compared with two other well-established guidelines, including 2015 ATA by Middleton et al. [18] with favorable comparison among the US reporting systems in predicting the malignancy risk.

**Table 1:** US patterns and estimated risk of malignancy proposed by American Thyroid Association ATA, 2015

Benign - Estimated risk of malignancy < 1%	
US features: Purely cystic nodules (no solid component)	
Very low suspicion risk - Estimated risk of malignancy < 3%	
US features: Spongiform or partially cystic nodules without any of US features defining low-, intermediate- or high-suspicion patterns	
Low suspicion risk - Estimated risk of malignancy 5–10%	
US features: Iso- or hyperechoic solid nodule, or partially cystic nodule with eccentric solid areas, without microcalcification, irregular margins, extrathyroidal extension (ETE) or taller-than-wide shape	
Intermediate suspicion risk - Estimated risk of malignancy 10–20%	
US features: Hypoechoic solid nodule with smooth margins without microcalcification, ETE or taller-than-wide shape	
High suspicion risk - Estimated risk of malignancy > 70–90%	
US features: Solid hypoechoic nodule or solid hypoechoic component of a partially cystic nodule with one or more of the following features: irregular margins (microlobulated, infiltrative), microcalcification, taller-than-wide shape, rim calcifications with small extrusive soft tissue component or ETE	

Few studies have addressed the role of US scores in indeterminate TNs [19–24]. Gao et al. [25] suggested that US is helpful for differentiating benign and malignant Bethesda III through a meta-analysis that included 2405 nodules. The specificity was 71% and sensitivity was 66% in nodules with 3 suspicious US features. The clinical significance of TI-RADS and 2015 ATA guidelines in the subcategorization of Bethesda III (AUS and FLUS) was estimated by Baser et al. [26] and Lee et al. [24], respectively. In both studies, malignant nodules in the AUS group had a significantly higher prevalence of suspicious US features than benign nodules. He et al. investigated the diagnostic performance of TI-RADS and a new US scoring system defined according to the risk score that was calculated based on marked hypoechogenicity, taller-than-wide and absence of the halo sign in indeterminate TNs. When the two methods were analyzed (TI-RADS and the new US scoring) in combination, the performance was superior to the use of TI-RADS alone with sensitivity, specificity, positive predictive value (PPV), negative predictive value (NPV) and accuracy of 97.6, 48.5, 70.9, 94.1 and 76.2%, respectively [27]. In this scenario, we believe that the US patterns should not only select the nodule for biopsy but also triage cytologically indeterminate TNs for US follow-ups, molecular analysis or surgery.

The combination of 2017 ACR TI-RADS or 2015 ATA with TBSRTC and its impact on the medical approach in indeterminate TNs has not been examined extensively. Our study aims to stratify the malignancy risk of indeterminate TNs (Bethesda III, IV and V) by combining the cytology and US features correlating with the final histopathology from 139 thyroidectomized patients.

## Methods

A retrospective observational study with a cohort registry of patients who underwent thyroid US, FNAC with indeterminate results and thyroidectomy was conducted after the Institutional Review Board Approval. Written informed patient consent was obtained for review of image and medical reports.

### Patients

The study was conducted at the Clinical Hospital of the Federal University of Paraná (HC-UFPR) in association with one external private institution in an iodine-replete area in Brazil. Initially, we performed a retrospective review on the database from our institution and from one external pathology center for all thyroid FNAC between January 2012 and June 2016. TNs were included in this study if they had (a) indeterminate cytology; (b) a thyroid US image and (c) surgical resection with a histopathological result matching with the nodule’s location and size analyzed on US-FNAC. Exclusion criteria were lack or absence of information on the US, FNAC or histology.

### Thyroid FNAC and cytological interpretation

**Fig. 1:**
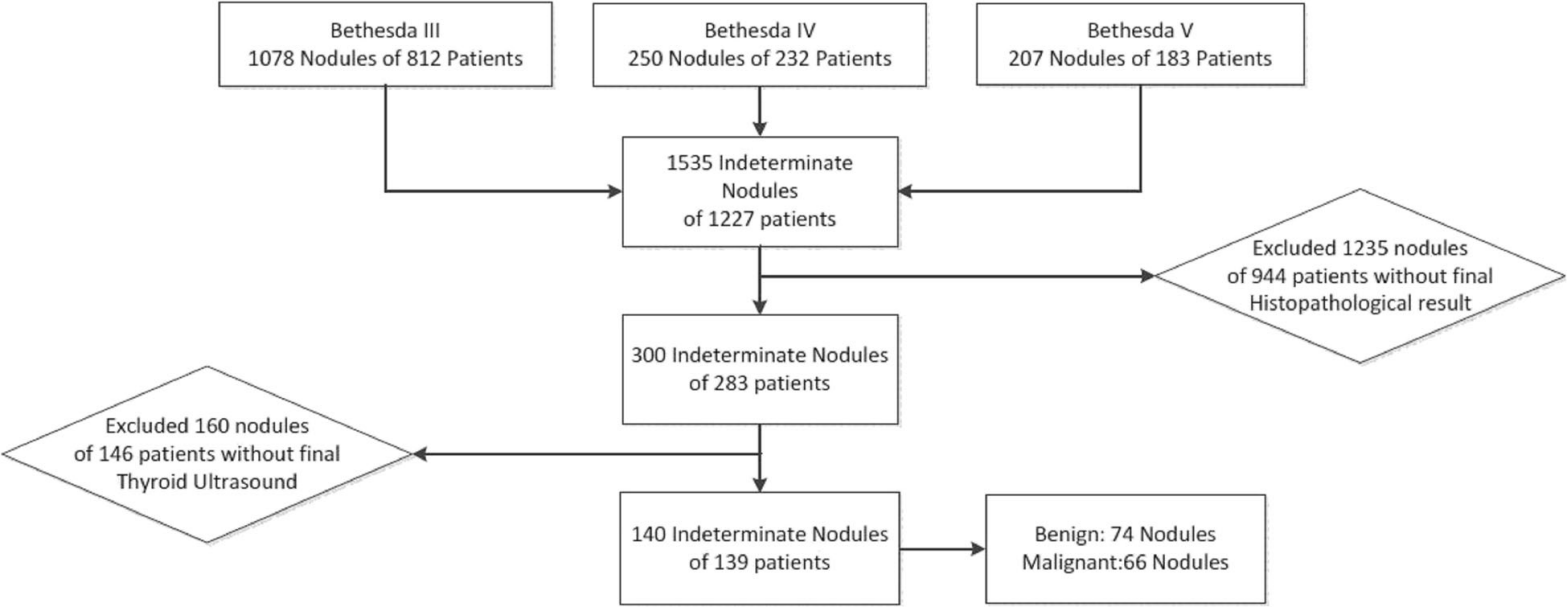
Diagram of study group

During the study period, 11.825 nodules were biopsied in 8.058 patients by experienced clinicians, cytopathologists or radiologists under US guidance. All nodules biopsied were classified according to the Bethesda system: 2.503 (21.1%) nondiagnostic (Bethesda I), 7.332 (62%) benign (Bethesda II), 1.078 (9.1%) AUS/FLUS (Bethesda III), 250 (2.1%) FN/SFN (Bethesda IV), 207 (1.7%) SM (Bethesda V) and 455 (3.9%) malignant (Bethesda VI) (Fig. 1).

### Histopathological results

The pathology reports pertaining to those patients who underwent surgery were searched at both pathology centers from our institution and the external private pathology center, and each nodule included in this study was considered benign or malignant for the diagnosis of thyroid cancer as our gold standard for data analysis. The lack of final histopathology was due to the loss of clinical and outpatient follow-up and difficulty in retrieving the histological results from external pathology centers. Incidentally thyroid cancer (microcarcinoma found in another nodule) was not considered for statistical analysis. The cytologic and histopathological evaluation were reviewed by an expert pathologist.

### Ultrasound examinations

Pre-FNA thyroid US examinations were retrieved from the database of our institution and four private radiology centers. In total, 140 indeterminate thyroid nodules in 139 patients (one patient had two different nodules biopsied with indeterminate results) were included in this study. US features of each enrolled nodule were reviewed and recorded in our study database by one expert radiologist who was blinded regarding the cytological and histological diagnoses. Microsoft Excel 2010 was the platform used for data collection and analysis. Information about the nodules’ size and location were provided to the radiologist. From static grayscale US images recorded during the original examination, the following categories were evaluated: composition, echogenicity, shape, margins and echogenic foci. Composition was classified as spongiform, mixed or solid. None of the purely cystic nodules were included in this analysis. The nodule was considered hyper-, iso- or hypoechoic to the surrounding thyroid parenchyma or as showing marked hypoechogenicity when compared with the adjacent strap muscle. Shape was classified as wider-than-tall or taller-than-wide (greater in the anteroposterior dimension than in the transverse dimension). Margins were classified as smooth, lobulated or irregular and if there was extrathyroidal extension (ETE). Echogenic foci, if present, were classified as macrocalcifications, peripheral (rim) calcifications and microcalcifications. Additionally, the presence of cervical lymphadenopathy was evaluated. US findings that were considered in favor of malignancy were hypoechoic or markedly hypoechoic echogenicity; irregular margins; presence of microcalcifications; taller-than-wide shape; evidence of ETE and the presence of lymphadenopathy.

### ACR TI-RADS and ATA classifications

Based on the 2015 ATA guidelines [2], the TNs were classified according to the malignancy risk as “high”, “intermediate”, “low” or “very low” suspicion (Table 1). Nine nodules in our study with iso- or hypoechoic echogenicity with one suspicious US feature were classified in the high suspicion category. For the TI-RADS classification, each US feature received point(s) according to the 2017 ACR TI-RADS publication [12], being classified as TR2 (2 points); TR3 (3 points); TR4 (4–6 points) and TR5 (≥ 7 points) (Table 2). In our study, the category TR4 was subdivided into TR4a (4 points), TR4b (5 points) and TR4c (6 points) due to its amplitude in the detection of nodules of higher or lower suspicion with scores varying from 4 to 6 points. None of the nodules were classified as “benign” by ATA or TR1 by ACR TI-RADS.

**Table 2:** American College of Radiology Thyroid Imaging Reporting and Data System (ACR TI-RADS) Classification

Ultrasound features		Description and points		
Composition (choose 1)		Cystic or almost completely cystic - 0 points		
		Spongiform - 0 points		
		Mixed cystic and solid - 1 point		
		Solid or almost completely solid - 2 points		
Echogenicity (choose 1)		Anechoic – 0 points		
		Hyperechoic or isoechoic – 1 point		
		Hypoechoic – 2 points		
		Very hypoechoic – 3 points		
Shape (choose 1)		Wider-than-tall – 0 points		
		Taller-than-wide – 3 points		
Margins (choose 1)		Smooth – 0 points		
		Ill-defined – 0 points		
		Lobulated or irregular – 2 points		
		Extra-thyroidal extension – 3 points		
Echogenic foci		None or large comet-tail artifacts – 0 points		
(choose all that apply)		Macrocalcifications – 1 point		
		Peripheral (rim) calcifications – 2 points		
		Punctate echogenic foci – 3 points		
TI-RADS Category (sum of points)				
TR1 (0 points) Benign	TR2 (2 points) Not Suspicious	TR3 (3 points) Mildly suspicious	TR4 (4–6 points) Moderately suspicious TR4a – 4 points TR4b – 5 points TR4c – 6 points	TR5 (≥7 points) Highly suspicious

ACR TI-RADS modified from Tessler et al.

### Statistical analysis

The patients’ ages and nodule size were described by their means and/or medians with standard deviation and/or range. Logistic regression models and Wald tests were used to evaluate factors associated with benign/malignant thyroid nodules. For statistical analysis, ACR TI-RADS 2, 3 and 4a nodules, “very low”, “low” and “intermediate suspicion” nodules by ATA guidelines and Bethesda III were considered favorable / negative while ACR TI-RADS 4b, 4c and 5 nodules, “high suspicion” nodules and Bethesda IV and V were considered unfavorable / positive, according to the likelihood of negative or positive results for malignancy. The level of concordance between the two ultrasound classifications (ACR TI-RADS and 2015 ATA) was estimated by the Kappa coefficient with a 95% confidence interval (CI). Sensitivity, specificity, positive predictive value (PPV), negative predictive value (NPV) and accuracy were calculated for the combination of Bethesda and US classifications. The statistical significance was set at *p* < 0.05. Statistical software (IBM SPSS Statistics v.20.0. Armonk, NY: IBM Corp) were used to conduct the data analysis.

## Results

The cohort comprised 44 (31.4%) Bethesda III, 52 (37.1%) Bethesda IV and 44 (31.4%) Bethesda V TNs identified in 139 patients (118 females), with a mean age of 49 ± 13 years, who had undergone thyroidectomy. Seventy-four (52.9%) of the 140 nodules were histologically diagnosed as benign (42 cases of nodular hyperplasia, 31 cases of follicular adenomas and 1 case of noninvasive follicular thyroid neoplasm with papillary-like features [NIFTP]). The 66 (47.1%) nodules that were malignant included 44 papillary thyroid cancers (PTC), 12 follicular-variant papillary thyroid cancers (FVPTC), 1 Warthin-like papillary carcinoma of the thyroid, 4 medullary thyroid cancers, 4 follicular thyroid carcinomas and 1 poorly differentiated thyroid carcinoma. The overall distribution in the TI-RADS and ATA categories was as follows: 5 TR2 (3.6%), 43 TR3 (30.7%), 32 TR4a (22.9%), 9 TR4b (6.4%), 23 TR4c (16.4%), 28 TR5 (20.0%), 5 “very low” (3.6%), 43 “low” (30.7%), 36 “intermediate” (25.7%) and 56 “high” (40.0%) suspicion nodules.

We initially evaluated the role of the patient’s age at diagnosis, sex, size of the lesion and US features in predicting malignancy. No significant association was observed for age at diagnosis, sex, or composition between the benign and malignant lesions (Table 3). However, the size of the lesion (*p* = 0.049), (marked) hypoechogenicity (*p* = 0.012), taller-than-wide nodule shape (*p* = 0.001), irregular margins (*p* < 0.001), and microcalcification (p < 0,001) were associated with nodule malignancy (Table 3).

**Table 3:** Association of clinical and ultrasound (US) features with final histopathological result of 140 nodules with indeterminate diagnosis

	Final histopathological results			
Clinical and US features	Benign (*n* = 74)	Malignant (*n* = 66)	P* value	OR (CI 95%)
*Age (yr)*				
Mean	49,6 ± 14,0 (18–84)	48,6 ± 13,3 (25–77)	0.663	0.99 (0.97–1.02)
*Gender*				
Female	64 (54.2%)	54 (45.8%)		
Male	9 (42.9%)	12 (57.1%)	0.339	1.58 (0.61–4.07)
*Size (cm)*	2.0 (0.72–8.33)	1.43 (0.54–7.57)	0.049	0.58 (0.33–0.99)
*Composition*				
Solid (ref)	67 (52.3%)	61 (47.7%)		
Mixed	4 (57.1%)	3 (42.9%)	0.738	1.37 (0.22–8.59)
Spongiform	3 (60%)	2 (40%)	0.921	1.13 (0.11–11.8)
*Echogenicity*				
Isoechoic (ref)	10 (71.4%)	4 (28.6%)		
Hyperechoic	28 (65.1%)	15 (34.9%)	0,664	1.34 (0.35–5.06)
Hypoechoic	25 (53.2%)	22 (46.8%)	0,232	2.20 (0.60–8.12)
Markedly hypoechoic	11 (30.6%)	25 (69.4%)	0,012	5.68 (1.44–22.4)
*Shape*				
Wider-than-tall (ref)	72 (60.0%)	48 (40.0%)		
Taller-than-wide	2 (10.0%)	18 (90.0%)	0.001	13.5 (2.94–61.9)
*Margins*				
Regular	66 (69.5%)	29 (30.5%)		
Lob/irregular	8 (17.8%)	37 (82.2%)	< 0.001	10.5 (4.32–25.6)
*Echogenic foci*				
Absent	70 (60.9%)	45 (39.1%)	-	
Macrocalcifications	0 (0%)	3 (100%)		
Peripheral calcification	1 (50%)	1 (50%)		
Microcalcifications	3 (15%)	17 (85%)		–
*Echogenic foci*				
Yes	4 (16.0%)	21 (84.0%)		
No	70 (60.9%)	45 (39.1%)	< 0.001	8.17 (2.60–25.7)

Results presented as mean ± standard deviation (minimum-maximum) or median (minimum-maximum); frequency (percent)

*Logistic regression models and Wald test; *p* < 0.05

*OR* odd ratios, *CI* confidence interval

The distribution of carcinomas among Bethesda categories III, IV and V was 15.9, 40.4 and 86.4%, respectively (p < 0.001) (Table 4). We next evaluated whether the US classifications are associated with lesion malignancy and found a positive correlation between the TI-RADS score and ATA guideline patterns, individually or pooled, and risk of malignancy. The rate of malignancy increased according to the suspicion on US (p < 0.001) (Table 4).

**Table 4:** Malignancy rate according to TBSRTC, ACR TI-RADS score and 2015 ATA pattern in 140 indeterminate thyroid nodules

Classification		Final Diagnosis				
	Number of cases	Benign (n = 74)	Malignant (n = 66)	Risk of malignancy (%)	P* value	OR (CI 95%)
*TBSRTC*						
Bethesda III	44	37	7	15.9%		
Bethesda IV	52	31	21	40.4%		
Bethesda V	44	6	38	86.4%	< 0.001	
*TBSRTC groups*						
III	44	37	7	15.9%		
IV/V	96	37	59	61.5%	< 0,001	8.43 (3.37–21.1)
*ACR TI-RADS*						
TR2	5	4	1	20.0%		
TR3	43	33	10	23.3%		
TR4a	32	23	9	28.1%		
TR4b	9	4	5	55.6%		
TR4c	23	8	15	65.2%		
TR5	28	2	26	92.9%	< 0.001	
*ACR TI-RADS groups*						
TR2/TR3/TR4a	80	60	20	25.0%		
TR4b/TR4c/TR5	60	14	46	76.7%	< 0.001	9.86 (4.46–21.8)
*2015 ATA patterns*						
Very low	5	4	1	20.0%		
Low	43	33	10	23.3%		
Intermediate	36	27	9	25.0%		
High	56	10	46	82.1%	< 0.001	
*2015 ATA groups*						
Very low / low / intermediate	84	64	20	23.8%		
High	56	10	46	82.1%	< 0.001	14.7 (6.24–34.7)

*Logistic regression models and Wald test; *p* < 0.05

*Abbreviations*: *ACR TI-RADS* American College of Radiology Thyroid Imaging Reporting and Data System, *ATA* American Thyroid Association, *CI* confidence interval, *OR* odd ratios, *TBSRTC* The Bethesda System for Reporting Thyroid Cytopathology

The diagnostic performance of ACR TI-RADS when considering category TR2/3/4a as negative and TR4b/4c/5 as positive was as follows: sensitivity, 69.7%; specificity, 81.1%; positive predictive value (PPV), 76.7%; negative predictive value (NPV), 75.0% and accuracy, 75.7%. The diagnostic performance of 2015 ATA when considering “very low”, “low”, or “intermediate” as negative and “high” as positive was as follows: sensitivity, 69.7%; specificity, 86.5%; PPV, 82.1%; NPV, 76.2% and accuracy, 78.6%. The kappa coefficient was estimated at 0.91 with a 95% confidence interval of k, 0.84–0.98, which was considered as ‘substantial’ agreement between TI-RADS and ATA.

In an attempt to stratify indeterminate lesions according to the risk of malignancy, we combined the cytology with 2017 ACR TI-RADS and 2015 ATA guidelines. The distribution of malignancy of the ACR TI-RADS score and 2015 ATA US patterns restricted to Bethesda III versus Bethesda IV and Bethesda V is shown in Table 5. Of the patients classified as Bethesda III and presenting a favorable ultrasound appearance (TI-RADS 2, 3 or 4a / ATA very low, low or intermediate), only 5.9 and 5.7% (TI-RADS and ATA, respectively) had malignant histological results. In contrast, 81.6 and 87.2% (TI-RADS and ATA, respectively) classified as Bethesda IV or V, which presented an ultrasound considered unfavorable (TI-RADS 4b, 4c and 5 / ATA high suspicion) had malignant histological results. In the discrepant groups, that is, favorable cytology (Bethesda III) and unfavorable ultrasound or unfavorable cytology (Bethesda IV or V) and favorable ultrasound the risk of malignancy was intermediate (Table 5).

**Table 5:** Distribution of malignancy of ACR TI-RADS score and 2015 ATA US patterns for Bethesda III vs IV and V categories in 140 indeterminate thyroid nodules

TBSRTC		2017 ACR TI-RADS score			2015 ATA US patterns		
		TR2 / TR3 / TR4a	TR4b / TR4c / TR5	Total	Very low / Low / Intermediate	High	Total
Bethesda III							
Histological Result	B	32	5	37	33	4	37
		94.1%	50.0%		94.3%	45.5%	
	M	2	5	7	2	5	7
		5.9%	50.0%		5.7%	55.5%	
Total		34	10	44	35	9	44
Bethesda IV and Bethesda V							
Histological Result	B	28	9	37	31	6	37
		40.4%	18.4%		63.3%	12.8%	
	M	19	40	59	18	41	59
		59.6%	81.6%		36.7%	87.2%	
Total		47	49	96	49	47	96

Histological Result: *B* benign and *M* malignant

To verify the diagnostic performance in the correlation between TBSRTC with ACR TI-RADS and 2015 ATA in predicting malignant anatomopathological (AP) results we calculated the parameters of sensitivity, specificity, PPV, NPV and accuracy using Bethesda III and 2/3/4a by TI-RADS or “very low”, “low”, “intermediate” by ATA as favorable / negative and Bethesda IV or V and 4b/4c/5 by TI-RADS or “high” by ATA as unfavorable / positive. For correlation between Bethesda and ACR TI-RADS, the parameters were as follows: sensitivity, 95.3% (89.1–100%); specificity, 78% (65.4–90.7%); PPV, 82% (71.4–92.6%); NPV, 94.1% (86.2–102%) and accuracy, 86.9% (79.7–94.1%). For correlation between Bethesda and 2015 ATA, the parameters were as follows: sensitivity, 95.3% (89.1–100%); specificity, 84.6% (73.3–95.9%); PPV, 87.2% (77.7–96.8%); NPV, 94.3% (86.6–102%) and accuracy, 90.2% (83.8–96.7%).

The percentage of malignancy in the final AP was estimated according to the US classifications (ACR TI-RADS [2 / 3 / 4a; 4b / 4c / 5] and 2015 ATA [“very low”, “low”, “intermediate”, or “high”]) and Bethesda system (III; IV/V) by logistic regression model and Wald test statistical analysis. The lowest US suspicion categories (TR2/3/4a and “very low”, “low”, or “indeterminate”) in Bethesda III had the highest likelihood of benignity, while the likelihood of malignancy in the final AP result increased progressively according to the increase in US suspicion and the Bethesda diagnostic categories (Table 6).

**Table 6:** Percentage of malignant anatomopathology (AP) combining Bethesda with ATA and ACR TI-RADS in 140 indeterminate thyroid nodules

ATA	Bethesda	Percentage of malignant AP
Very low / low / intermediate	III	7.00%
Very low / low / intermediate	IV/V	35.80%
High	III	50.40%
High	IV/V	88.20%
ACR TI-RADS		
2/3/4a	III	8.10%
2/3/4a	IV/V	37.50%
4b/4c/5	III	42.50%
4b/4c/5	IV/V	83.50%

## Discussion

The differentiation of benign and malignant lesions among cytologically indeterminate TNs has represented a challenge in clinical practice. As the overall rate of malignancy is low in indeterminate TNs, a high rate of benignancy is expected after surgery and a tool that helps to rule out thyroid cancer preoperatively might reduce the number of diagnostic surgeries and, hence, the risks involved in this procedure [28]. According to the results of our study, the combination of US classifications, ACR TI-RADS and ATA, with the Bethesda system is useful for detecting benign lesions in Bethesda III nodules and malignant lesions in Bethesda IV/V nodules. Both US classifications were effective in ruling out malignancy in Bethesda III TNs with low-risk US categories: “very low”, “low” and “intermediate” - ATA and TR2, TR3 and TR4a - ACR TI-RADS (NPV 94.1% from ACR TI-RADS and 94.3% from ATA) while indeterminate Bethesda IV/V TNs with high-risk US categories: “high” – ATA and TR4b, TR4c and TR5 – TI-RADS had the highest risk of malignancy (88.2 and 83.5%, respectively). The NPV obtained in the Bethesda III category was quite similar to the results obtained using molecular tests [6, 7, 29, 30], reinforcing the conclusion that US classification may be helpful for decision-making in favor of a conservative approach and follow-up of nodules in this category.

Several studies in recent years have suggested the use of US patterns to stratify the risk of malignancy of indeterminate TNs [19–23, 25, 31, 32]. According to the results of our study, Grani et al. analyzed the accuracy of TI-RADS and ATA to stratify malignancy risk in 49 indeterminate TNs, concluding that nodules classified as TI-RADS 3 or very low suspicion could be conducted conservatively (NPV 100% when the estimated risk of malignancy for the test positivity was set at 3%). The PPV for intermediate and high suspicion of ATA and TI-RADS 4c was 63 and 71%, respectively [19]. Maia et al. studied the combination of the TI-RADS score with the Bethesda system to stratify malignancy risk in 136 indeterminate TNs, showing high sensitivity (80%) and NPV (90%) in nodules classified as Bethesda III and TI-RADS 3 and 4a scores, implying a conservative approach in these cases. In contrast, nodules scored as TI-RADS 4b and 5 with Bethesda IV and V presented with a higher risk of malignancy (75 and 76.9%, respectively) [20]. Rocha et al. prospectively analyzed 137 patients with 143 indeterminate TNs (Bethesda III and IV) who were referred for surgery. Considering noninvasive follicular thyroid neoplasms with papillary-like nuclear features (NIFTP) and tumors of uncertain malignant potential (TUMP) as benign, the rate of malignancy was 72, 22.4, 4.3, 0, and 15.4% for nodules with high suspicion, intermediate suspicion, low suspicion, very low suspicion, and undefined ultrasonographic pattern, respectively [33]. Most of the studies used one of the TIRADS classifications, but few used the ACR TI-RADS. Few studies analyzed the 2015 ATA guidelines in indeterminate TNs, and the majority used small cohorts with conflicting results [19, 21, 24, 34].

Cytologically indeterminate nodules had a malignancy risk implied in each category as follows: 10–30% in Bethesda III, 25–40% in Bethesda IV and 50–75% in Bethesda V [3, 5]. The percentage of each Bethesda category (III, IV and V) from the total of thyroid FNACs performed in the period of study was consistent with the literature [9, 35], presenting a chance of 13% of a thyroid FNAC having an indeterminate result. Even Bethesda III, which has wide variability in its use due to the difficulty of defining specific criteria, presents within the limit of interpretation to 7–10% of all FNACs [5]. Despite the large number of losses we had from the initial group of patients as a result of the lack of US examination and histological results, we believe that our sample was representative since the malignancy risk estimated in each Bethesda category was consistent with the literature.

In relation to the US classifications (TI-RADS and ATA), the estimated risk of malignancy of each US category was comparable to studies that specifically analyzed indeterminate TNs, since these nodules presented with a risk of malignancy higher than that found in the nodules in general (40% versus 5 to 10%). Chaigneau et al. analyzed 602 indeterminate TNs classified as TI-RADS score 3, 4a, 4b and 5 with the following malignancy risks: 20.5, 29, 63.4 and 100%, respectively [36]. For ATA US patterns, Tang et al. classified 49 indeterminate TNs according to 2015 ATA guidelines with the following malignancy risk: “very low” (12%), “low” (17%), “intermediate” (21%) and “high” (100%) [34]. Among nodules classified by ATA in our study, the malignancy risks were consistent with the literature for “low suspicion” (23.3%), “intermediate” (25%) and “high suspicion” (82.1%) patterns; however, the malignancy risk for “very low” (20.0%) was higher than that found in the literature. For thyroid nodules classified by ACR TI-RADS, the malignancy risk for each score was similar to the study previously discussed: TR3 (23.3%), TR4 (the mean of the three categories - TR4a, TR4b and TR4c: 49.6%) and TR5 (92.9%). Due to the variability of cancer risk observed in our study for TR4 score (4a – 28.1%; 4b – 55.6% and 4c – 65.2%), we subdivided this category because we observed that TR4a had a behavior more similar to that of low risk, while TR4b and 4c had a behavior similar to that of high risk. Among the 5 nodules classified as “very low suspicion” and TR2 in our study, only one nodule demonstrated malignant histology, but in this case, the Bethesda category was SM, which is a category of higher cancer risk. It is noteworthy that malignancy rates increased proportionally to the risk of cancer in each category. Interestingly, from nine nodules with iso- or hypoechoic echogenicity that presented with one high suspicion US feature, such as microcalcifications, irregular margins or taller-than wide shape and were classified as having a “high” suspicion of malignancy in our study, eight ultimately had a malignant histology. Valderrabano et al. evaluated 463 indeterminate TNs (AUS/FLUS and FN/SFN) and concluded that there were no differences in the prevalence of malignancy between nodules with “low” (iso/hyperechoic) or “intermediate” (hypoechoic) suspicion patterns, concluding that hypoechogenicity alone did not seem to improve the risk stratification of indeterminate TNs [37]. In contrast, any suspicious US feature significantly increases the risk of malignancy of indeterminate TNs. In the same way, we identified in our study that the intermediate suspicious nodules of ATA had a behavior more similar to those of low risk than high risk.

The diagnostic performance of TI-RADS and ATA, in our study, was evaluated by the sensitivity, specificity, PPV, NPV and accuracy for each US classification and the comparison between the two US systems by the calculation of the Kappa concordance coefficient, which showed ‘substantial’ agreement between 2017 ACR TI-RADS and 2015 ATA guidelines, being equivalent to the evaluation of the cytologically indeterminate TNs by both classifications in clinical practice. The malignancy risk progressively rises among the ACR TI-RADS categories whereas in 2015 ATA there is only one important difference between the intermediate and the high suspicion (the very low, low and intermediate suspicions behave very similarly). To the best of our knowledge, our study is the first to evaluate and compare the performance of the ACR TI-RADS and the 2015 ATA guidelines exclusively on indeterminate TNs.

Despite the many US classifications that have been investigated [13, 18, 38–42], surgery is still often necessary for the diagnosis. Some US features are described as suspicious for several studies [12, 14–17], such as nodule hypoechogenicity, irregular margins, microcalcifications and taller-than-wide shape, although the diagnostic accuracy of individual US features is insufficient for surgical decision-making [43]. The analysis of these characteristics in our study is consistent with the above studies. Although the reinterpretation of the US is not recommended for the decision-making after indeterminate cytology [43], the results of our study suggested that US features may be considered to guide the clinical management of indeterminate TNs.

There were several limitations in our study. The main limitation was the loss bias of the sample, noted once we searched for patients with analyzable ultrasound examinations, and had significant problems in the final studied group due to the difficulty of retrieving thyroid US images and histological results. Despite this setback, we believe that our sample was representative once the prevalence of malignancy in each category of Bethesda (III, IV and V) is similar to the literature. In addition, the high NPV in Bethesda III with low-risk US categories may be relied upon in a service where the risk of malignancy is comparable with our study. If the service has higher risk of malignancy in this category, the correlation may not be reliable. Finally, there was a lack of information about some clinical parameters known to be associated with thyroid cancer, such as thyroid autoimmunity and TSH level, which deserve to be tested to evaluate their impact on the performance of the US.

## Conclusions

In conclusion, the ACR TI-RADS score and the 2015 ATA US patterns can refine the malignancy risk assessment, suggesting a conservative approach for indeterminate Bethesda III TNs with low-risk US categories (“very low”, “low” and “intermediate” and TR2, TR3 and TR4a), surgical approach for Bethesda IV and V with high-risk US categories (“high” and TR4b, TR4c and TR5) and molecular testing or diagnostic surgery for Bethesda III with high-risk US features or Bethesda IV and V with low-risk US features.

## Data Availability

The datasets used and/or analysed during the current study are available from the corresponding author on reasonable request.

## Abbreviations

ACR: American College of Radiology
AP: Anatomopathological
ATA: American Thyroid Association
AUS/FLUS: Atypia of undetermined significance/follicular lesion of undetermined significance
FN/FSN: Follicular neoplasm/suspicious for a follicular neoplasm
FNAC: Fine-needle aspiration cytology
NPV: Negative predictive value
PPV: Positive Predictive Value
SEMPR: Serviço de Endocrinologia e Metabologia do Paraná
SM: Suspicious for malignancy
TBSRTC: The Bethesda System for Reporting Thyroid Cytopathology
TI-RADS: Thyroid imaging reporting and data system
TNs: Thyroid nodules
US: Ultrasound
